# Morphological Characterization and Failure Analysis of the Ultrasonic Welded Single-Lap Joints

**DOI:** 10.3390/polym15173555

**Published:** 2023-08-26

**Authors:** Quanyue Zhao, Hantai Wu, Xinyu Chen, Xiaoxuan Chen, Shuaiheng Xu, Chunwang He, Tian Zhao

**Affiliations:** 1 Institute of Advanced Structure Technology, Beijing Institute of Technology, Beijing 100081, China; 3120185021@bit.edu.cn (Q.Z.);; 2 State Key Laboratory of Explosion Science and Technology, Beijing Institute of Technology, Beijing 100081, China

**Keywords:** ultrasonic welding, thermoplastic composites, damage mechanics, finite element analysis (FEA)

## Abstract

Ultrasonic welding technology represents an advanced method for joining thermoplastic composites. However, there exists a scarcity of systematic investigations into welding parameters and their influence on the morphological characteristics and quality of the welded regions. Furthermore, a comprehensive experimental understanding of the welded joint failure mechanisms remains deficient. A robust model for simulating the failure behavior of welded joints under loading has yet to be formulated. In this study, ultrasonic welded specimens were fabricated using distinct welding control methods and varied parameter combinations. Diverse experimental methodologies are employed to assess the morphological features of the welded areas, ascertain specimen strength, and observe welding interface failure modes. Based on a cohesive model, a finite element model is developed to predict the strength of the ultrasonic welded joints and elucidate the failure mechanisms. The results showed that, under identical welding parameters, the specimens welded with a high amplitude and low welding force exhibit superior welding quality. The specimens produced under displacement control exhibit minimal dispersion in strength. The proposed finite element model effectively prognosticates both welded joint strength and failure modes.

## 1. Introduction

In the current milieu characterized by the rigorous regulation of carbon emissions, a substantial influx of lightweight materials, such as aluminum alloy, magnesium alloy, and polymeric composites, has been incorporated into key manufacturing sectors [1,2,3]. Among various lightweight materials, thermoplastic composites (TPCs) stand out due to their recyclability, cost-effective manufacturing, and high damage tolerance, and have gained increasing attention from the aerospace and automotive industries [4]. Given the capability of TPCs to undergo a phase transition from a molten state at elevated temperatures to a solid state upon cooling, TPCs can be welded through fusion bonding [5]. Among the various welding techniques, ultrasonic welding stands out as a highly promising method for TPC assembly, which can be attributed to its advantages, such as its high energy efficiency, the rapid welding process, the absence of foreign materials at the weldline, and its potential for in-situ process monitoring [6].

Presently, a plethora of research on the ultrasonic welding of thermoplastic composites has been disseminated, with a predominant focus on the heating mechanisms [7,8] and optimization of the welding procedure [9,10,11,12,13]. Villegas [14] employed a microprocessor-controlled ultrasonic welder to analyze the transformations and heating mechanisms at the welded interface and their correlation with dissipated power and sonotrode displacement. This enabled the direct monitoring of the welding process and assessment of weld quality through the feedback provided by the ultrasonic welder, thereby establishing a robust foundation for subsequent experimental research and the broader application of ultrasonic welding. Based on this, she discerned a progressive enhancement in weld strength aligned with the power curve, culminating at the zenith during the second power plateau, corresponding to Stage 4 of the entire process. The study also revealed that compared to time-controlled or energy-controlled welding, an advantage of displacement-controlled welding is its relatively diminished sensitivity to fluctuations in the welding force and vibration amplitude [15]. Wang et al. [16] examined the weld attributes in the ultrasonic welding of short carbon fiber reinforced PA6 under various welding conditions. They identified three different failure modes of the joints, representing distinct weld attributes. The bonding efficiency and weld area increased with the higher welded energy until reaching a threshold. Levy et al. [17,18] performed a series of numerical studies for facilitating the understanding of the ultrasonic welded process. A level-set method was proposed to simulate the flow of EDs during the melting phase [19], and the effects of typical process parameters, e.g., vibration amplitudes, holding force, and adherend thicknesses, on the welded process were analyzed [20]. With the advancement of artificial intelligence (AI), some studies have employed AI techniques to predict the strength of welded joints [21]. Li et al. [22] identified eight welding characteristics during the welding process and, using artificial intelligence methods, established a relationship between these welding characteristics and welding strength, thereby achieving the objective of predicting the strength of the welded specimens.

However, to date, there has been a paucity of systematic research examining the impact of various welding parameter combinations on welding quality. Moreover, the methodologies to assess welding quality are rather limited, predominantly focusing on strength testing and cross-sectional analysis [15,16]. Notably, cross-sectional analysis presents certain drawbacks: firstly, it is a destructive technique, and during the sectioning of specimens, the intrinsic morphological features might be compromised; and secondly, it provides insights only into the characteristics of the welding interface at a specific cross-section, devoid of any holistic view. Additionally, the failure mechanisms of the welding interface are primarily deciphered through SEM observations of the fracture surface [23,24,25]. The understanding of the damage evolution process at the welding interface during load application remains scant.

This study systematically investigated the variations in the strength, appearance, and failure modes of single-lap specimens welded using different parameters and control methods. Concurrently, non-destructive CT scanning technology was employed to characterize the entire welded surface, analyzing the disparities in the welding interface morphology arising from different welding parameters and control techniques. Additionally, finite element models were developed considering both intralaminar damage within the composite layers and damage at the welding interface to analyze the failure mechanisms and damage evolution of the single-lap welded joints under tensile load.

## 2. Experimental

### 2.1. Material

**Figure 1:**
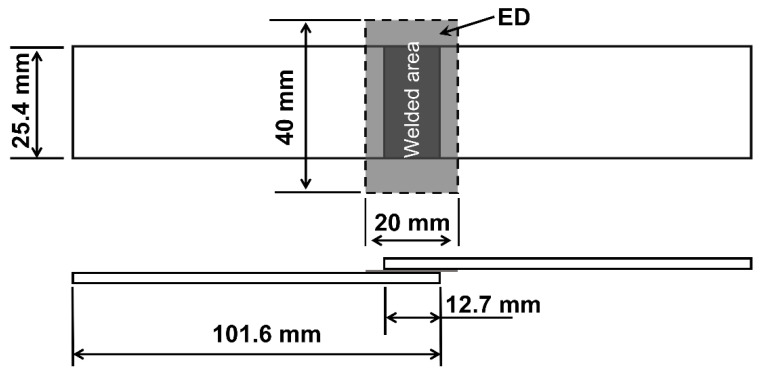
Schematic of the single-lap weld specimen (Dimensions in mm).

PEEK demonstrates a high thermal resistance and, upon exposure to fire, produces notably low levels of smoke and toxic emissions, making it particularly valuable in aerospace and other critical applications [26]. Therefore, the unidirectional carbon fiber-reinforced PEEK composite laminates with 66 wt.% carbon fiber, manufactured by Junhua PEEK Company, were employed in this study. The stacking sequence of the laminate is [0°/90°]_4S_. The thickness of the consolidated laminates was 2 ± 0.01 mm (average ± standard deviation, measured at 6 locations). The mechanical properties of the laminates are listed in Table 1. Rectangular adherends with a size of 101.6 × 25.4 mm^2^ were cut from these laminates with a water-cooled diamond saw according to the standard ASTM D1002, as shown in Figure 1. The energy directors (EDs) used in this study were flat PEEK films, manufactured by Junhua PEEK Company, with a nominal thickness of approximately 0.45 mm. The PEEK films were cut into the size of 20 × 40 mm^2^, which was slightly larger than the welded area, to completely cover the overlapping surface.

### 2.2. Ultrasonic Welding

**Figure 2:**
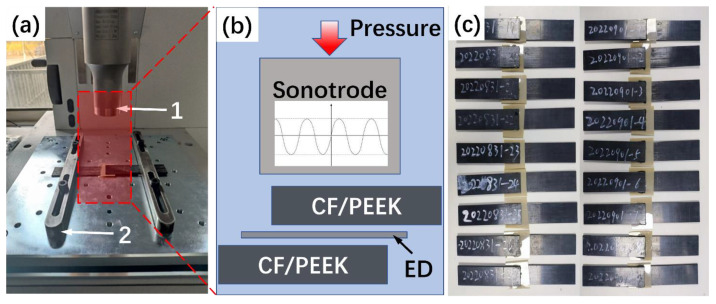
(**a**) Ultrasonic welder and welding jigs used in this study: 1. circular sonotrode with a diameter of 40 mm, 2. welding jig for DLS specimens; (**b**) schematic representation of the welding stacking sequence; (**c**) welded specimens.

An ultrasonic welder (HiQ DIALOG S, 20 kHz, 4800 W), produced by Herrmann Ultrasonics, was employed to perform the welding of the specimens. Figure 2a shows the welding set-up equipped with a 40 mm-diameter cylindrical sonotrode and custom-made welding jigs. The welding stacking sequence is depicted in Figure 2b. Flat energy directors, which were made of PEEK, the same material as the matrix in TPC, were used to concentrate heat generation at the welding interface.

In order to investigate the effects of different welding control methods and welding parameters on the quality of welded specimens, based on our prior experience, we selected three sets of welding parameters: high amplitude (HA)-low force (LF) (40 μm-500 N), high amplitude (HA)-high force (HF) (40 μm-1500 N), and low amplitude (HA)-high force (LF) (25 μm-1500 N). For each set of welding parameters, we utilized three different control methods: displacement-controlled welding, energy-controlled welding, and time-controlled welding. 

**Figure 3:**
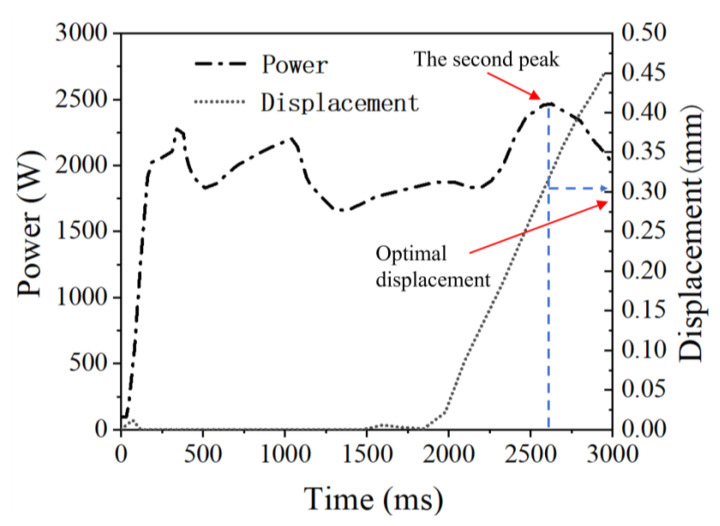
Power and displacement curves for 100% welding displacement.

As mentioned in Reference [15], the welding quality is optimal when the welding power curve reaches the second peak, which is the fourth stage of the welding process. Therefore, for the welding parameter set HA-LF, at the very beginning, we employed displacement-controlled welding with a welding displacement of 0.45 mm (100% ED thickness, namely entire welding travel) to achieve the optimal welding displacement. By analyzing the welding process curve obtained from the recorded data, as shown in Figure 3, we identified the optimal welding displacement (0.3 mm) corresponding to the second peak in the power curve. Displacement-controlled welding has the advantage of being relatively insensitive to fluctuations in the welding force and the vibration amplitude, unlike time-controlled or energy-controlled welding. Therefore, we believe that 0.3 mm is the optimal welding displacement for the welding parameter sets HA-HF and LA-HF.

**Figure 4:**
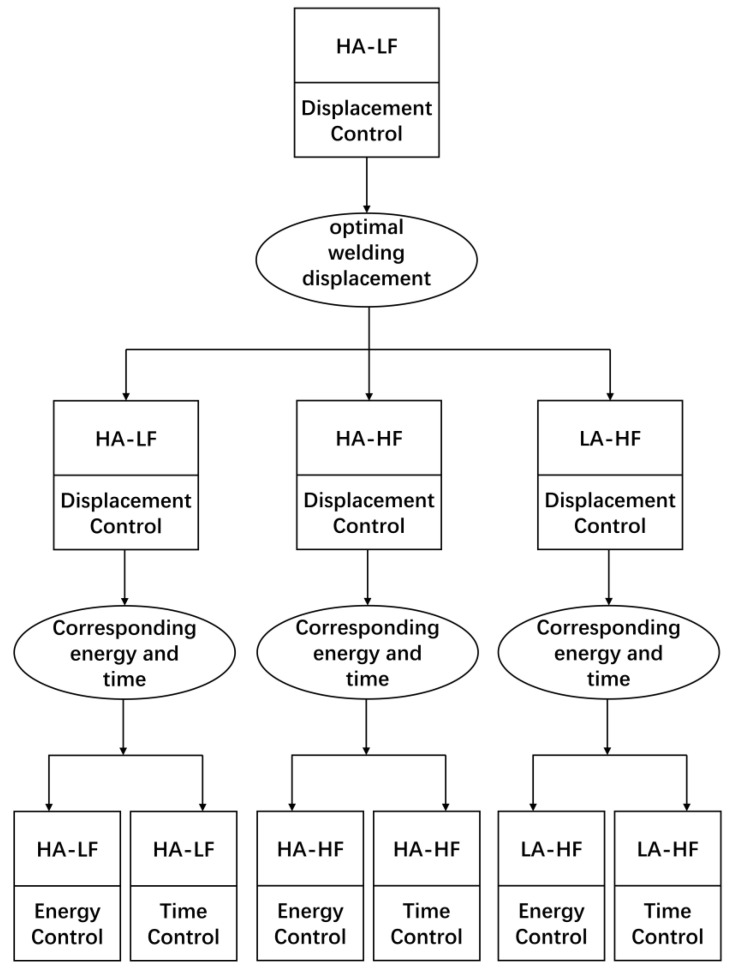
Flowchart of the welding experiment process.

Subsequently, we performed three welding trials using a displacement-controlled welding of 0.3 mm with these welding parameter sets and recorded the welding energy and welding time for each trial. The average values of welding energy and welding time were then calculated and used as controlling parameters for energy-controlled welding and time-controlled welding. The welding parameters and controlling parameters for the entire welding experiment are listed in Table 2. The entire welding experiment procedure is shown in Figure 4. In total, 36 specimens were obtained from the welding process. These specimens were used to investigate the influence of different control modes and welding parameters on the quality of the welded joints.

### 2.3. Micro-CT and Mechanical Tests

XWT-240-CT imported from Vaux, Germany, which has a 225 KV and 2000 μA X-ray tube and minimum voxel sizes of up to 4 μm, was adopted to conduct the μCT scan test. Three specimens, which were welded with displacement-controlled welding, were selected for each set of welding parameters for the μCT scan test. The welded zone in each specimen was scanned with an X-ray source voltage of 170 kV and current of 100 μA. During each scan, 1000 projections were acquired in 60 min. The whole sample was reconstructed using VG studio software and the effective voxel size of the CT images was 15 μm. The detailed 3D tomographic images were processed by Avizo 2019 software to evaluate the morphological characteristics of the welded joint.

All specimens were subjected to mechanical testing according to the experimental standards ASTM D1002 to obtain their strength values. The tests are carried out using a ZWICK 100 kN universal test machine with self-aligning wedge grips. The cross-head speed is set to 1 mm/min and the test is automatically stopped after measuring a load drop to 75% of the highest measured force. The ambient temperature during the mechanical test was 25 °C, and the relative humidity was 60%. The length of the specimen clamped at both ends is 25.4 mm. To eliminate the influence of geometric asymmetry in the specimen, aluminum sheets of the same thickness as the substrate are fixed and adhered to both ends of the specimen using epoxy resin. After mechanical tests, the specimens welded using displacement-controlled welding with a different set of welding parameters were subjected to cross-sectional observations to examine the morphology of the fracture surfaces using Scanning Electron Microscope (SEM) and determine the failure modes of the specimens.

## 3. Modeling Method

### 3.1. Continuous Damage Model for the TPC Adherends

At the mesoscale, a unidirectional fiber-reinforced TPC (UD-TPC) was employed as an equivalent homogenized layer with linear elastic and orthotropic properties within a laminate configuration. The initiation of damage was determined by employing the three-dimensional Hashin’s criterion [29]. Furthermore, a linear damage evolution law was integrated, considering the dissipation of fracture energy during the damage progression. The evolution of each damage variable was defined using equivalent strain, which was specifically tailored to account for the tension and compression failure modes of both the fibers and matrix. The model was implemented in the commercial FE code Abaqus/explicit framework via VUMAT subroutine written as a Fortran script.

#### 3.1.1. Constitutive Relationship of UD-TPC Lamina

In this research, the single layered UD-TPC lamina was treated as linear elastic and orthotropic material. The 3D constitutive equation for a UD-TPC lamina is given by Equation (1). The term ($C_{ij}^{}$) in Equation (1) represents the stiffness of the lamina without damage [30]. This term can be mathematically expressed in terms of measurable parameters, including Young’s modulus and Poisson’s ratio, as described by Equation (2). After the initiation of damage, the material stiffness undergoes degradation throughout the damage evolution process. The constitutive relationship, incorporating the updated (degraded) stiffness terms, is described by Equation (3).

(1) $$\{\begin{array}{c} \sigma_{11}\\ \sigma_{22}\\ \sigma_{33}\\ \sigma_{13}\\ \sigma_{23}\\ \sigma_{31} \end{array}\}=\left[\begin{array}{cccccc} C_{11} & C_{12} & C_{13} & 0 & 0 & 0\\ C_{12} & C_{22} & C_{23} & 0 & 0 & 0\\ C_{13} & C_{23} & C_{33} & 0 & 0 & 0\\ 0 & 0 & 0 & 2G_{12} & 0 & 0\\ 0 & 0 & 0 & 0 & 2G_{23} & 0\\ 0 & 0 & 0 & 0 & 0 & 2G_{31} \end{array}\right]\{\begin{array}{c} \varepsilon_{11}\\ \varepsilon_{22}\\ \varepsilon_{33}\\ \varepsilon_{12}\\ \varepsilon_{23}\\ \varepsilon_{31} \end{array}\}$$

(2) $$\begin{array}{l} C_{11}=\frac{\left(1-\nu_{23}\nu_{32}\right)}{E_{2}E_{3}\Delta };C_{12}=\frac{\left(\nu_{21}+\nu_{23}\nu_{31}\right)}{E_{2}E_{3}\Delta };C_{13}=\frac{\left(\nu_{31}+\nu_{21}\nu_{32}\right)}{E_{2}E_{3}\Delta };\\ C_{23}=\frac{\left(\nu_{32}+\nu_{23}\nu_{31}\right)}{E_{1}E_{3}\Delta };C_{22}=\frac{\left(1-\nu_{13}\nu_{31}\right)}{E_{1}E_{3}\Delta };C_{33}=\frac{\left(1-\nu_{12}\nu_{21}\right)}{E_{1}E_{2}\Delta };\\ \Delta =\frac{1-\nu_{12}\nu_{21}-\nu_{23}\nu_{32}-\nu_{13}\nu_{31}-2\nu_{21}\nu_{32}\nu_{13}}{E_{1}E_{2}E_{3}} \end{array}$$

(3) $$\{\begin{array}{c} \sigma_{11}\\ \sigma_{22}\\ \sigma_{33}\\ \sigma_{13}\\ \sigma_{23}\\ \sigma_{31} \end{array}\}=\left[\begin{array}{cccccc} C_{11}^{d} & C_{12}^{d} & C_{13}^{d} & 0 & 0 & 0\\ C_{12}^{d} & C_{22}^{d} & C_{23}^{d} & 0 & 0 & 0\\ C_{13}^{d} & C_{23}^{d} & C_{33}^{d} & 0 & 0 & 0\\ 0 & 0 & 0 & 2G_{12}^{d} & 0 & 0\\ 0 & 0 & 0 & 0 & 2G_{23}^{d} & 0\\ 0 & 0 & 0 & 0 & 0 & 2G_{31}^{d} \end{array}\right]\{\begin{array}{c} \varepsilon_{11}\\ \varepsilon_{22}\\ \varepsilon_{33}\\ \varepsilon_{12}\\ \varepsilon_{23}\\ \varepsilon_{31} \end{array}\}$$

The term ($C_{ij}^{d}$) in Equation (3) indicates the stiffness of the lamina with damage. It can be computed with the undamaged stiffness ($C_{ij}$) and the damage variables ($d_{f},d_{m}$, and $d_{s}$) given by Equation (4). $d_{f},d_{m}$, and $d_{s}$ represent the damage variables for fiber, matrix, and shear damage, respectively. They can be derived by Equations (5)–(7).

(4) $$\begin{array}{l} C_{11}^{d}=(1-d_{f})C_{11};C_{12}^{d}=(1-d_{f})(1-d_{m})C_{12};C_{13}^{d}=(1-d_{f})(1-d_{m})C_{13};\\ C_{22}^{d}=(1-d_{m})C_{22};C_{33}^{d}=(1-d_{m})C_{33};C_{23}^{d}=(1-d_{f})(1-d_{m})C_{23};\\ G_{12}^{d}=(1-d_{s})G_{12};G_{23}^{d}=(1-d_{s})G_{23};G_{13}^{d}=(1-d_{s})G_{13} \end{array}$$

(5) $$d_{f}=1-\left(1-d_{ft}\right)\left(1-d_{fc}\right)$$

(6) $$d_{m}=1-\left(1-d_{mt}\right)\left(1-d_{mc}\right)$$

(7) $$d_{s}=1-\left(1-d_{ft}\right)\left(1-d_{fc}\right)\left(1-d_{mt}\right)\left(1-d_{mc}\right)$$

where the damage variables $d_{ft},d_{fc},d_{mt}$, and $d_{mc}$ indicate the fiber tension damage mode, fiber compression damage mode, matrix tension damage mode, and matrix compression damage mode, respectively. The evaluation of these four damage variables is conducted utilizing a damage evolution law.

#### 3.1.2. Damage Initiation Criteria

The failure within the welded joints at the optimal condition includes intra-laminar failures that occur within the composite structure itself, such as fiber breakage and resin rupture, which are primarily observed at the outermost layer of the TPC adherends.

In this study, the Hashin damage criterion, which incorporates four representative failure modes for unidirectional fiber-reinforced composite materials, namely fiber breakage in tension/compression and matrix damage in tension/compression, was employed to characterize the intra-lamina fracture behavior of the TPC adherends. The corresponding governing equations are expressed as follows:

Fiber breakage in tension ($\sigma_{11}>0$):

(8) $$F_{ft}={\left(\frac{\sigma_{11}}{X_{t}}\right)}^{2}+\frac{\sigma_{12}^{2}+\sigma_{13}^{2}}{S_{12}^{2}}=\{\begin{array}{c} \geq 1failute\\ \leq 1no^{}\;failure \end{array}$$

Fiber breakage in compression ($\sigma_{11}<0$):

(9) $$F_{fc}={\left(\frac{\sigma_{11}}{X_{c}}\right)}^{2}=\{\begin{array}{c} \geq 1failure\\ \leq 1no^{}\;failure \end{array}$$

Matrix damage in tension ($\sigma_{22}+\sigma_{33}\geq 0$):

(10) $$F_{mt}={\left(\frac{\sigma_{22}+\sigma_{33}}{Y_{t}}\right)}^{2}+\frac{\sigma_{23}^{2}-\sigma_{22}\sigma_{33}}{S_{23}^{2}}+\frac{\sigma_{12}^{2}+\sigma_{13}^{2}}{S_{12}^{2}}=\{\begin{array}{c} \geq 1failure\\ \leq 1no^{}\;failure \end{array}$$

Matrix damage in compression ($\sigma_{22}+\sigma_{33}<0$):

(11) $$F_{mc}=\left[{\left(\frac{Y_{c}}{2S_{23}}\right)}^{2}-1\right]\left(\frac{\sigma_{22}+\sigma_{33}}{Y_{c}}\right)+{\left(\frac{\sigma_{22}+\sigma_{33}}{2S_{23}}\right)}^{2}-\frac{\sigma_{23}^{2}-\sigma_{22}\sigma_{33}}{S_{23}^{2}}+\frac{\sigma_{12}^{2}-\sigma_{13}^{2}}{S_{12}^{2}}=\{\begin{array}{c} \geq 1failure\\ <1no_{}\;failure \end{array}$$

where $\sigma_{ij}$ represents the components of the effective stress tensor, $X_{t}$ and $X_{c}$ represent the tensile and compressive strength in the longitudinal direction, $Y_{t}$ and $Y_{c}$ represent the tensile and compressive strength in the transverse direction, $S_{12}$, $S_{13}$, and $S_{23}$ are the in-plane and out-of-plane shear strengths in different directions, respectively.

#### 3.1.3. Damage Evolution

This section focuses on the post-damage initiation behavior of the UD-TPC lamina. Prior to any damage initiation, the lamina exhibits linear elastic behavior and follows the constitutive relationship described by Equation (1). However, once damage initiation takes place, the stiffness of the lamina begins to degenerate under the influence of subsequent loading. This degradation is accounted for by updating the stiffness matrix, which is then incorporated into the constitutive relationship expressed by Equation (3). The degradation in stiffness is governed by the damage variables assigned to the lamina, as defined in Equations (5)–(7). To determine the values of the damage variables, a local damage variable is assigned to each of the four damage modes and evaluated using Equation (12) [30]. These local damage variables range between zero (representing an undamaged state) and one (representing a fully damaged state).

(12) $$d_{i}=\frac{\varepsilon_{eq}^{f,i}\left(\varepsilon_{eq}^{i}-\varepsilon_{eq}^{o,i}\right)}{\varepsilon_{eq}^{i}\left(\varepsilon_{eq}^{f,i}-\varepsilon_{eq}^{o,i}\right)}\left(i=ft,\;fc,\;mt,\;mc\right)$$

where $\varepsilon_{eq}^{o,i}$ and $\varepsilon_{eq}^{f,i}$ represent the equivalent strains at damage initiation and fracture, respectively. The introduction of strain-softening leads to pronounced mesh dependency due to strain localization, resulting in a decrease in energy dissipation as the mesh is refined. To address this issue, linear strain softening is adopted during the damage evolution, which is based on the energy dissipation ($G_{c}^{i}$) approach proposed by Hillerborg et al. [31]. The energy dissipated during the fracture of an element is directly proportional to the size of the element [32]. To address the issue of mesh dependency, the fracture energies are normalized with the characteristic length of the elements ($L_{c}$). It is assumed in the model that the energy dissipation during crack propagation is equivalent to the critical strain energy release rate during damage. The energy dissipation corresponding to each of the four damage modes within an element is calculated using Equation (13).

(13) $$\int \sigma_{eq}^{i}d\left(\varepsilon_{eq}^{i}L_{c}\right)=G_{c}^{i}\left(i=ft,\;fc,\;mt,\;mc\right)$$

where $\sigma_{eq}^{i},\;\varepsilon_{eq}^{i},\;L_{c}$, and $G_{c}^{i}$ indicate the equivalent stress, equivalent strain, and characteristic length of an element, and the fracture energy of each failure mode. For linear stiffness degradation, Equation (13) takes a simple form to calculate $\varepsilon_{eq}^{f,i}$ using Equations (14)–(17). Further, the equivalent strains $\varepsilon_{eq}^{i}$ and $\varepsilon_{eq}^{o,i}$ in Equation (12) under the 3D state of stress are also computed using Equations (14)–(17).

Fiber tension:

(14) $$\varepsilon_{eq}^{ft}=\sqrt{{\langle \varepsilon_{11}\rangle }^{2}+\varepsilon_{12}^{2}+\varepsilon_{13}^{2}};\varepsilon_{eq}^{o,ft}=\frac{X_{1t}}{E_{1}};\varepsilon_{eq}^{f,ft}=\frac{2G_{c}^{ft}}{X_{1t}L_{c}}$$

Fiber compression:

(15) $$\varepsilon_{eq}^{fc}=-\langle \varepsilon_{11}\rangle ;\varepsilon_{eq}^{o,fc}=\frac{X_{1c}}{E_{1}};\varepsilon_{eq}^{f,fc}=\frac{2G_{c}^{fc}}{X_{1c}L_{c}}$$

Matrix tension:

(16) $$\varepsilon_{eq}^{mt}=\sqrt{{\langle \varepsilon_{22}\rangle }^{2}+{\langle \varepsilon_{33}\rangle }^{2}+\varepsilon_{12}^{2}+\varepsilon_{13}^{2}};\varepsilon_{eq}^{o,mt}=\frac{X_{2t}}{E_{2}};\varepsilon_{eq}^{f,mt}=\frac{2G_{c}^{mt}}{X_{2t}L_{c}}$$

Matrix compression:

(17) $$\varepsilon_{eq}^{mt}=\sqrt{{\langle -\varepsilon_{22}\rangle }^{2}+{\langle -\varepsilon_{33}\rangle }^{2}+\varepsilon_{12}^{2}+\varepsilon_{13}^{2}};\varepsilon_{eq}^{o,mc}=\frac{X_{2c}}{E_{2}};\varepsilon_{eq}^{f,mc}=\frac{2G_{c}^{mc}}{X_{2c}L_{c}}$$

where 〈x〉 denotes the Macauley operator, which can be described as:

(18) $$\langle x\rangle =\frac{1}{2}\left(x+\left|x\right|\right)$$

### 3.2. Cohesive Model for the Welding Interface

**Figure 5:**
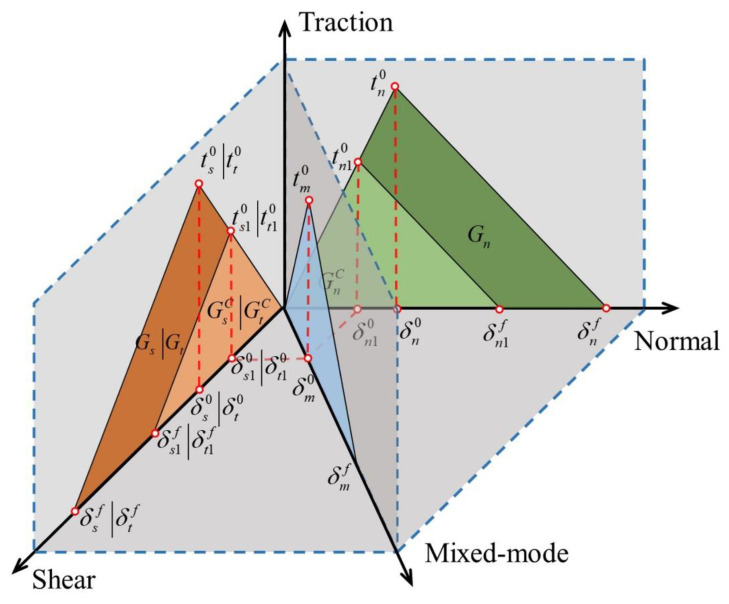
The bilinear traction-separation law in CZM [37].

To model the failure behavior of the welding interface, the cohesive zone model (CZM), which is widely employed for characterizing fracture behavior in composite interfaces [33,34,35], was utilized in this research. To achieve a balance between simplicity and accuracy, a bilinear traction-separation law, as shown in Figure 5, was chosen to simulate the progressive damage of the TPC welded joints [36].

To predict the initiation of damage in the welded areas, specifically in the cohesive zone, the quadratic traction initiation criterion was employed. This criterion, expressed mathematically as follows, enables the determination of the critical conditions for damage initiation [38]:

(19) $$\sqrt{{\left(\frac{\langle t_{n}\rangle }{t_{n}^{0}}\right)}^{2}+{\left(\frac{t_{s}}{t_{s}^{0}}\right)}^{2}+{\left(\frac{t_{t}}{t_{t}^{0}}\right)}^{2}}=1$$

where $t^{0}$ represents the peak value of the traction force. 

The onset of damage is hypothesized to occur when the value of the quadratic traction function reaches 1. The material stiffness is degraded once the damage initiation criterion is satisfied and gradually reduces to zero, which indicates the final failure of the joint.

The Benzeggagh–Kenane (BK) mixed-mode failure criterion was adopted to simulate the damage evolution of the cohesive interface, which is expressed as follows [39]:

(20) $$G^{C}=G_{n}^{C}+\left(G_{s}^{C}+G_{n}^{C}\right){\left(\frac{G_{s}+G_{t}}{G_{n}+G_{s}+G_{t}}\right)}^{\eta }$$

where $G_{n}^{C}$, $G_{s}^{C}$, and $G_{t}^{C}$, which are characterized by the areas under the traction-displacement curves in Figure 5, refer to the critical fracture energies required to cause failure in the respective directions. Meanwhile, η is an empirical parameter which is extracted from the mixed mode I/II test [40].

### 3.3. FE Model

**Figure 6:**
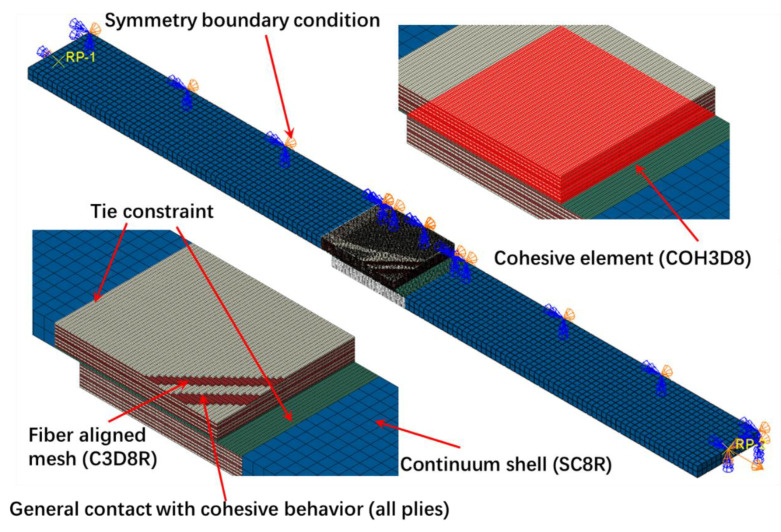
High-fidelity FE model of the single-lap joint under tensile load.

A high-fidelity 3D finite element (FE) model to simulate both the welding interface behavior and intra-lamina behavior of the welded single-lap joint under tensile load was built in the ABAQUS 2019 dynamic explicit version, as shown in Figure 6. To enhance computational efficiency, a half-domain finite element model was established, and subsequently, the computational results of the entire model were obtained through the implementation of mirroring techniques.

The laminate at the welded zone was set up by using a ply-by-ply modeling method where each ply is discretized as a layer of reduced-integration solid elements (C3D8R). Each layer is connected through general contact with cohesive behavior. Following the modeling guidelines proposed in Reference [41], the fiber-aligned mesh with an aspect ratio of 2.4 is constructed in the welded zone. The composite support sections, which are far from the welded joint, are discretized with through-thickness continuum shell elements (SC8R). Only linear elastic properties were assigned, and no strength failure criteria were employed to simulate this section. The connection between the damage zone and support sections is implemented with a “tie” constraint in order to transfer displacements and rotation. All the related parameters of the composite laminate, including the parameters for the Hashin criterion, were listed in Table 1. Zero-thickness cohesive elements (COH3D8) were inserted between the two adherends at the welded joint to simulate the welding interface. The related cohesive parameters are summarized in Table 3.

During the loading process, the left end of the lower adherend was fully clamped, whereas the right end of the upper adherend was fixed at the y- and z-directions. The symmetry boundary condition was applied to one side of the specimen. A displacement of 1 mm was applied at the right end of the upper adherend along the x-direction.

## 4. Results and Discussion

### 4.1. Experimental Results

#### 4.1.1. Mechanical Test

**Figure 7:**
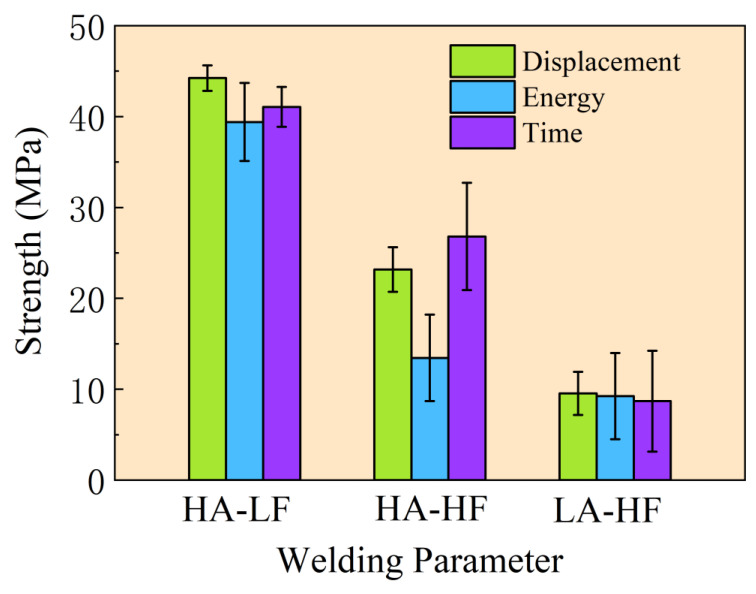
Statistical analysis of tensile strength.

Figure 7 presents the tensile test results of the specimens welded with different welding parameters and control methods. According to the test results, it is evident that the specimens welded with HA-LF parameters exhibited superior mechanical strength. Specifically, the average tensile strength of the thermoplastic composite joints welded with the welding parameters of HA-LF was the highest, with an average value of 41.57 MPa. On the other hand, the specimens welded with LA-HF parameters exhibited the lowest tensile strength, with an average value of 9.21 MPa.

Regarding different control methods, as depicted in Figure 7, it can be observed that regardless of the welding parameters, the specimens welded with displacement-controlled welding exhibited the smallest dispersion in mechanical strength. Specifically, the dispersion of the specimens welded with displacement-controlled welding, under the parameters of HA-LF, was the smallest, measuring 3.17%. This result is consistent with the findings presented in Reference [15]. Conversely, the specimens welded with energy-controlled welding exhibited the highest dispersion. The strength dispersion of the specimens welded with energy-controlled welding, under the parameters of LA-HF, was the largest, measuring 51.28%. Therefore, based on the results for the single-lap joint welding procedure, the optimal parameter set for thermoplastic composite welding is HA-LF, and displacement control is the best control method.

#### 4.1.2. Morphological Characterization of the Welding Interface

Three specimens, which were welded with the displacement control method, were selected for each set of welding parameters to conduct the μCT scan test. The threshold segmentation technique in Avizo software was employed to segment different components in the CT scan images obtained. It should be noted that due to the similar density of carbon fiber and PEEK, the fibers and matrix in the welded region cannot be distinguished in the images obtained by the CT scan. Therefore, the threshold segmentation technique can only distinguish the voids from the fiber-matrix region in the welding area.

**Figure 8:**
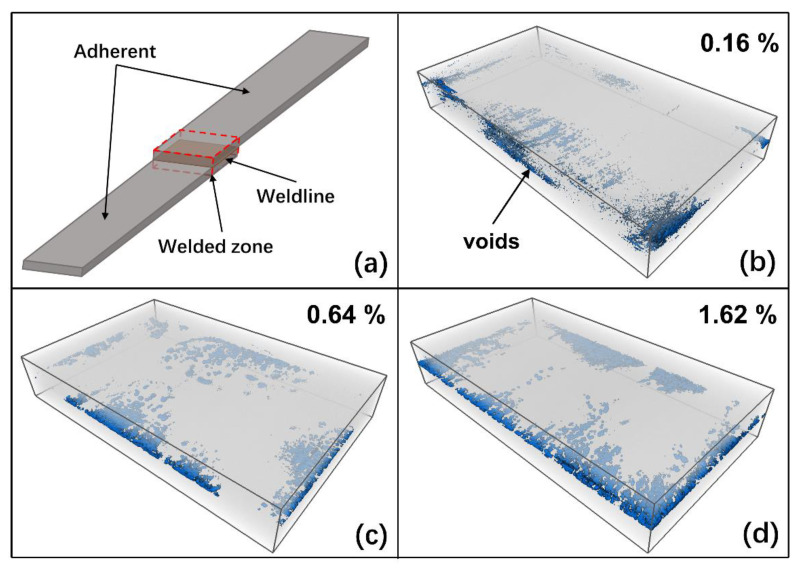
Reconstruction of the void at the welding interface: (**a**) specimen (**b**) HA-LF; (**c**) HA-HF; (**d**) LA-HF.

The CT images were segmented, especially the voids at the welding interface. Then the voids were then reconstructed, and the porosity was calculated, as shown in Figure 8. It can be seen that the voids are mainly distributed along the edges of the welding interface. The comparison between the porosity at the welding interface and the tensile strength of the specimens reveals a significant correlation between the mechanical strength of the specimen and porosity at the welding interface. As the porosity decreases, the welding strength gradually increases. The specimens welded using HA-LF parameters exhibited the lowest porosity at the welding interface, measuring 0.16%, and the average volume of the voids is relatively small, with the highest tensile strength. On the other hand, the specimens welded using LA-HF parameters had the highest porosity at the welding interface, measuring 1.42%, and the average volume of the voids is relatively large, resulting in the lowest tensile strength. The presence of voids diminishes the integrity of the welding interface, resulting in a reduction in the welded joint strength. This demonstrates the critical role of void presence at the welding interface in the failure process of the welded interface. This conclusion is consistent with the findings presented in Reference [43].

#### 4.1.3. Fracture Surfaces Characterization

**Figure 9:**
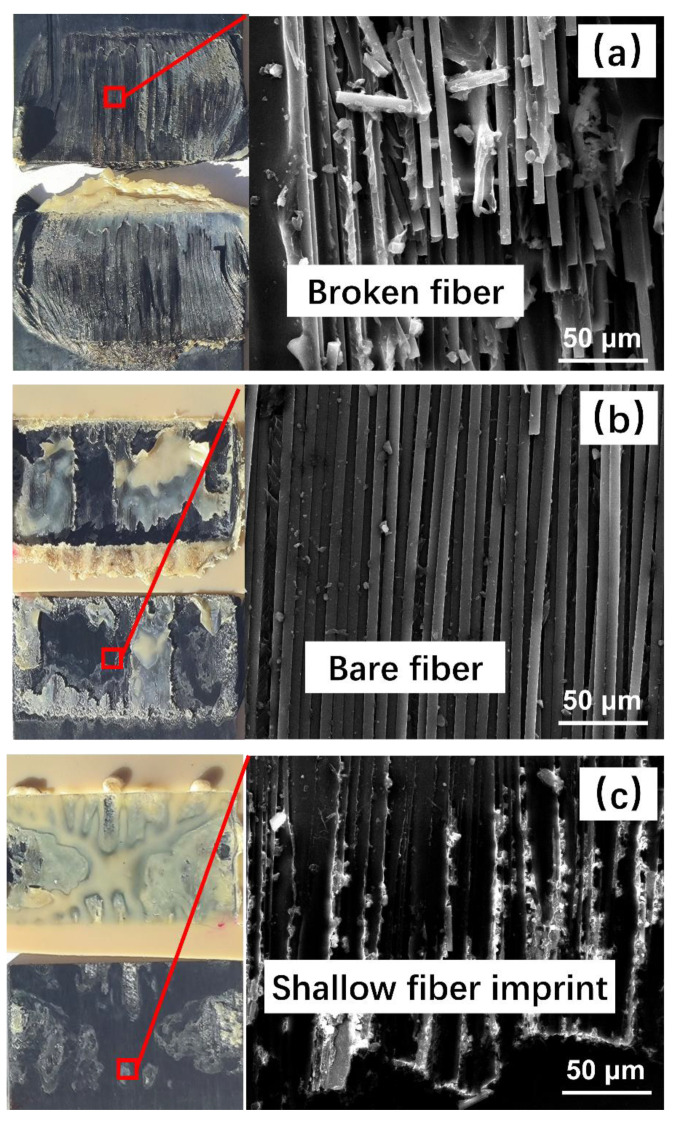
Observation of the fracture surface: (**a**) HA-LF; (**b**) HA-HF; (**c**) LA-HF.

Fractographic analysis was performed after the mechanical tests to identify the failure mechanisms of the welded joints. The specimens welded with displacement-controlled welding and different welding parameters were observed using scanning electron microscopy (SEM) to examine the fracture surfaces. Figure 9 presents the macroscopic and microscopic morphology of the joint fracture surfaces after the tensile test.

In Figure 9a, the fracture surface morphology and SEM microstructure of the specimens welded using HA-LF parameters are shown. It can be discerned that the resin at the welding interface, both in the Energy Director (ED) and matrix of the substrate’s surface layer, has undergone complete fusion, accompanied by a noticeable deformation of the fibers proximate to the periphery of the welding region. The SEM microstructure reveals fiber fracture within the substrate, indicating that the fibers bear the load during the tensile process and contribute to the optimal welding strength.

In Figure 9b, the fracture surface morphology of the specimens welded using HA-HF parameters is depicted. It can be observed that there is a minor amount of unmelted ED at the welding interface. We hypothesize that the observed phenomenon is due to the high-amplitude vibrations initiating the melting of the thermoplastic matrix material at the edge of the welding interface. However, the high welding force causes the molten matrix to be rapidly displaced downwards by the welding head, achieving the target welding displacement in a brief duration, and leaving portions of the matrix within the welding interface unmelted. Such conditions result in the specimens not attaining the anticipated welding strength. SEM analysis of the fracture surface unveils exposed fibers, with individual fibers remaining intact, signifying that the failure mechanism at the welding interface is primarily due to the lack of adhesion between the fibers and matrix. This leads to the reduced welding strength exhibited by these specimens.

In Figure 9c, the fracture surface morphology of the specimens welded under LA-HF parameters is depicted. A substantial region of unmelted thermoplastic matrix material is evident. We postulate that this arises due to the high melting point of PEEK, compounded by the inadequate power output of the low-amplitude equipment, making it challenging to attain the necessary temperature for PEEK melting. Consequently, effective cross-linking between the thermoplastic matrix and the substrate remains unachieved. This results in these specimens exhibiting a comparatively lower welding strength. SEM micrographs reveal superficial fiber imprints at the welding interface, suggesting a diminished interfacial bonding between the fibers and matrix, which contributes to the observed reduced welding strength.

### 4.2. Numerical Result

To simulate different welding qualities, two sets of material parameter values (case 1 and case 2) were chosen for cohesive elements in the calculation, as illustrated in Table 3. Case 1 simulates the specimen welded with HA-LF welding parameters, namely the optimal welded condition. Case 2 simulates the specimen welded with HA-HF and LA-HF welding parameters, namely under the welded condition. Due to factors such as slippage at the specimen grips and the deformation of the testing machine’s jaws, the displacement values measured during the experiments were significantly overestimated. Therefore, displacement cannot be used as a reliable measure to validate the effectiveness of the model. In this study, the validity of the model is assessed by comparing the strength obtained from the experiments with the calculated strength of the specimens.

**Figure 10:**
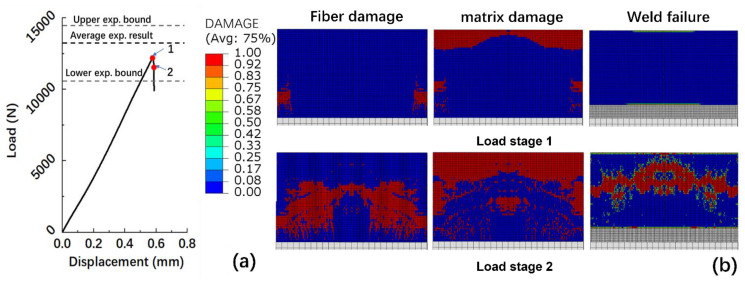
(**a**) Calculated load-displacement curve for case 1; (**b**) calculated failure mode for case 1.

As shown in Figure 10a, the ultimate load capacity of the optimal welded joint that the FE model calculated is 12,285 N, and the strength is 38 MPa, which is 8.4% lower than the average experimental data. The failure mode included fiber damage, matrix damage, and welded interface damage, which was consistent with the observations shown in Figure 9a. 

Load stage 1 is the welded joint damage initial point. At this load stage, the matrix at the free end of the first layer and the fiber at the fixed end of the first layer failed first, as shown in Figure 10b, while the welded interface did not fail. As the tensile displacement further increases, the failure of the matrix and fiber in the first layer of the substrate extends. Although the welded interface did not fail in the initial stage of damage, the matrix and fiber that bonded to the interface have already failed. As a result, the welding interface that bonded with the failed matrix and fiber cannot bear the load. Consequently, the area of the welded interface capable of bearing the load continually decreases, and eventually the load at the interface reaches the failure strength, resulting in the failure of the welding interface, as shown in Figure 10b. 

**Figure 11:**
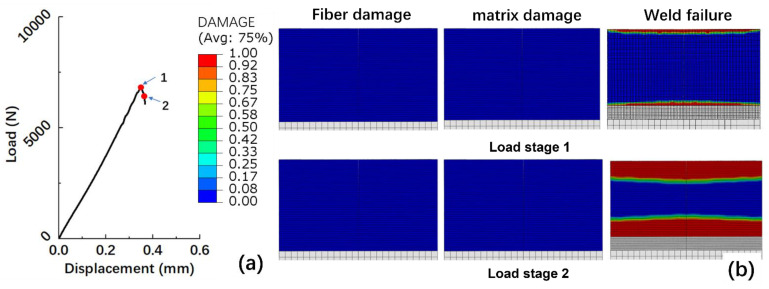
(**a**) Calculated load-displacement curve for case 2; (**b**) Calculated failure mode for case 2.

As shown in Figure 11a, the ultimate load capacity of the optimal welded joint that the FE model calculated is 6815 N, and the strength is 21 MPa. Only the welded interface failed during the loading process, which was consistent with the observations shown in Figure 9b,c. At the damage initial stage 1, the welded interface at the edge perpendicular to the load direction failed first, as shown in Figure 11b. As the tensile displacement increases, the area of interface failure gradually enlarges until the whole welded interface fails. Unlike in case 1, in this case, neither the fibers nor the matrix in the substrate undergo delamination throughout the entire process due to the lower strength of the welded interface. 

## 5. Conclusions

This investigation offers an in-depth exploration of the effects that diverse control strategies and welding parameters exert on the welding quality of thermoplastic composite joints via ultrasonic welding. In tandem with this, based on the theory of continuum damage mechanics, a sophisticated finite element model for a single-lap joint under tensile loading was constructed, facilitating a comprehensive analysis of its inherent failure mechanisms. Harnessing a synergistic blend of empirical evaluations and advanced computational analytics, the study has gleaned the following profound insights:

1. Under the same control method, welding with HA-LF pressure parameters resulted in better welding quality. the average tensile strength of the thermoplastic composite joints welded with the welding parameters of HA-LF was the highest, with an average value of 41.57 MPa. Among the control methods, displacement control achieved the lowest strength dispersion in the specimens, indicating a more stable and quality process compared to energy control and time control.
2. The strength of the welded joint was highly correlated with the porosity content at the welded interface. The presence of porosity affects the integrity of the welding joint, emphasizing the importance of minimizing void formation during the welding process as an effective means to improving welding quality.
3. A finite element model, grounded on a cohesive approach, was successfully established and adeptly predicted the strength and failure modes of ultrasonically welded joints. The failure mode of the welding joint was associated with the strength of the welding interface, i.e., the welding quality. When the welding interface had high strength, the failure involved fiber failure, matrix failure, and interface failure. In contrast, when the interface strength was low, the joint failure was primarily attributed to interface failure.

However, since the constructed model does not reflect the microscopic welding defects, it cannot accurately correlate welding quality with welding strength. In future work, multiscale finite element methods can be employed to simulate microscopic manufacturing defects, establishing a relationship between welding quality and welding strength. In addition, the mechanism of resin melting and deformation at the welding interface under the action of ultrasonic vibration and welding pressure requires further investigation through simulation calculations.

## Figures and Tables

**Table 1:** Material properties of the UD-TPC [27,28].

Property	Description	Value	Unit
E1	Young’s modulus in longitudinal direction	130,000	MPa
E2	Young’s modulus in transverse direction	15,000	MPa
G12	Out-of-plane shear modulus	4085	MPa
G23	In-plane shear modulus	3478	MPa
ν12	Poisson ratio	0.258	
ν23	Poisson ratio	0.286	
XT	Longitudinal tensile strength	2200	MPa
XC	Longitudinal compressive strength	1200	MPa
YT	Transverse tensile strength	100	MPa
YC	Transverse compressive strength	260	MPa
S12	Out-of-plane shear strength	107	MPa
S23	In-plane shear strength	40	MPa
$G_{c}^{ft}$	Fiber tensile fracture toughness	125	N/mm
$G_{c}^{fc}$	Fiber compressive fracture toughness	61	N/mm
$G_{c}^{mt}$	Matrix tensile fracture toughness	2	N/mm
$G_{c}^{mc}$	Matrix compressive fracture toughness	5	N/mm

**Table 2:** Welding control method and welding parameters for the welding process.

	HA-LF (40 μm-500 N)	HA-HF (40 μm-1500 N)	LA-HF (25 μm-1500 N)
Displacement (mm)	0.3	0.3	0.3
Energy (J)	1550	850	2160
Time (s)	2.4	0.38	1.17

**Table 3:** Material properties of the cohesive zone [28,42].

	Initial Stiffness (MPa)		Interfacial Strength (MPa)		Fracture Toughness (N/mm)	
	$K_{n}^{0}$	$K_{s}^{0}=K_{t}^{0}$	$\sigma_{n}^{0}$	$\tau_{s}^{0}=\tau_{t}^{0}$	$G_{n}^{C}$	$G_{s}^{C}=G_{t}^{C}$
Case 1	3800	3725	50	70	0.199	0.65
Case 2	3800	3725	30	50	0.199	0.65

## Data Availability

The data presented in this study are available on request from the corresponding author.
